# Sensitization of Glioma Cells to Tamoxifen-Induced Apoptosis by Pl3-Kinase Inhibitor through the GSK-3β/β-Catenin Signaling Pathway

**DOI:** 10.1371/journal.pone.0027053

**Published:** 2011-10-27

**Authors:** Cuixian Li, Chun Zhou, Shaogui Wang, Ying Feng, Wei Lin, Sisi Lin, Ying Wang, Heqing Huang, Peiqing Liu, Yong-Gao Mu, Xiaoyan Shen

**Affiliations:** 1 Laboratory of Pharmacology and Toxicology, School of Pharmaceutical Sciences, Sun Yat-sen University, Guangzhou, China; 2 Department of Histology and Embryology, Zhongshan School of Medicine, Sun Yat-sen University, Guangzhou, China; 3 Department of Neurosurgery/Neuro-oncology, Cancer Center, Sun Yat-sen University, Guangzhou, China; Indiana University School of Medicine, United States of America

## Abstract

Malignant gliomas represent one of the most aggressive types of cancers and their recurrence is closely linked to acquired therapeutic resistance. A combination of chemotherapy is considered a promising therapeutic model in overcoming therapeutic resistance and enhancing treatment efficacy. Herein, we show by colony formation, Hochest 33342 and TUNEL staining, as well as by flow cytometric analysis, that LY294002, a specific phosphatidylinositide-3-kinase (PI3K) inhibitor, enhanced significantly the sensitization of a traditional cytotoxic chemotherapeutic agent, tamoxifen-induced apoptosis in C6 glioma cells. Activation of PI3K signaling pathway by IGF-1 protected U251 cells from apoptosis induced by combination treatment of LY294002 and tamoxifen. Interference of PI3K signaling pathway by PI3K subunit P85 siRNA enhanced the sensitization of U251 glioma cells to tamoxifen -induced apoptosis. By Western blotting, we found that combination treatment showed lower levels of phosphorylated Akt^Ser473^ and GSK-3β^Ser9^ than a single treatment of LY294002. Further, we showed a significant decrease of nuclear β-catenin by combination treatment. In response to the inhibition of β-catenin signaling, mRNA and protein levels of Survivin and the other three antiapoptotic genes Bcl-2, Bcl-xL, and Mcl-1 were significantly decreased by combination treatment. Our results indicated that the synergistic cytotoxic effect of LY294002 and tamoxifen is achieved by the inhibition of GSK-3β/β-catenin signaling pathway.

## Introduction

Malignant gliomas are the most common primary tumors in the human brain. Despite the conventional treatments that include surgical resection, radiation therapy, and chemotherapy, frequent tumor recurrence results in poor prognosis with a mean survival time of 9–12 months for grade IV and 2 years for grade III glioma [1]. Exploring new therapeutic modes are, therefore, necessary for improving the outcomeof glioma treatment.

Tamoxifen, a potent estrogen receptor (ER) antagonist derived from nonsteroid triphenylethylene, has been extensively used to treat ER^+^ breast cancer. There have been increasing reports showing that a high dose of tamoxifen(4–8 fold higher than that for ER+ breast cancers) could be beneficial in the treatment of ER^−^ tumors, including glioma [2], [3]. However, in almost all the trials, only a subgroup of patients with malignant gliomas responded (response rate of about 30%) or were stabilized with prolonged treatment of high doses of tamoxifen [4], [5].

Although several intracellular signaling pathways, such as Protein kinase C, transforming growth factor-β, calmodulin, the transcription factor c-Myc, and the mitogen-activated protein kinases p38 and c-Jun NH_2_-terminal kinase have been implicated, the precise molecular mechanism of ER-independent proapoptotic activity of tamoxifen remains unclear [6], [7], [8], [9]. A better understanding of the mechanism of tamoxifen-induced apoptosis of malignant glioma cells would help improve the sensitivity and the response of the cells to tamoxifen treatment [10].

The phosphatidylinositide-3-kinase (PI3K)/Akt signaling pathway might be the best characterized pathway in the transmission of anti-apoptotic signals for cell survival. PI3K pathways regulate several malignant phenotypes including antiapoptosis, cell growth, and proliferation [11]. Activation of PI3K pathway is associated with poor prognosis in glioma patients [12]. Activated PI3K phosphorylates several downstream effectors including AKT. Akt is a critical mediator of PI3K signaling located at an intersection of multiple pathways involved in cell proliferation, survival, transcription, and metabolic processes [13], [14]. Inhibitors of PI3K and AKT have undergone pre-clinical evaluation with encouraging results. Perifosine, an oral AKT inhibitor, is undergoing clinical evaluation for malignant gliomas [11], [15]. However, most clinical trials of low molecular-weight kinase inhibitors as monotherapies have failed to demonstrate survival benefit in unselected malignant glioma patient populations. In response, combinations of targeted agents with radiation or chemotherapy may overcome therapeutic resistance and enhance treatment efficacy [11].

In this study, we compared the effect of a single treatment with LY294002, a specific phosphatidylinositide-3-kinase (PI3K) inhibitor, or tamoxifen, a traditional cytotoxic chemotherapic agent, with a combination treatment of the two agents on induction of apoptosis in C6 glioma cells. The underlying mechanisms are further explored in order to develop a novel and effective therapeutic approach against malignant gliomas.

## Materials and Methods

### Chemicals and Antibodies

DMSO, tamoxifen and LY294002 were purchased from Sigma-Aldrich,USA. Rabbit polyclonal antibodies against phospho-AKT(Ser473), AKT(pan), GSK-3β (pan), phospho-GSK-3β, β-catenin were purchased from Cell signal technology (USA). Horseradish peroxidase-conjugated secondary antibody was obtained from promega (USA); Alexa Fluor 488-conjugated goat anti-rabbit antibody was from Molecular Probes; Nuclear Extraction Kit was from Active Motif (USA); TUNEL Kit was from Beyotime (Jiangsu, China); Antifade Solution was from Applygen Technologies Inc (Beijing, China); Enhanced chemiluminescence (ECL) kit was from GE Healthcare (USA); EDTA-free protease inhibitor was from Roche.

### Cell treatment and plate colony formation assays

Rat C6 glioma cell (ATCC cat. number CCL-107), U251, and U87 purchased from the China Center for Type Culture Collection of Chinese Academy of Sciences were cultured in Dulbecco's modified Eagle's medium (DMEM) containing 10% fetal bovine serum, 100 Units/ml penicillin and 100 µg/ml streptomycin. Cells were seeded in 60 mm plates at a density of 5×10^5^ cells/ml and incubated overnight. The cells were serum-starved for 24 h and followed by treatment with LY294002 or/and tamoxifen. Control cells were incubated with vehicle only (DMSO) at a concentration equal to that in drug-treated cells (final concentration 0.1%). For combination experiments, quiescent cells were treated with LY294002 with additional tamoxifen at various concentrations at indicated times.

After 12 h treatment with LY294002, or/and tamoxifen, U251 cells were seeded into six-well plates (100 cells per well) and cultured for 14 days. The plates were stained with Giemsa, and the colonies were quantified. Each experiment was repeated in triplicate.

### Transfection of PI3K p85 siRNA and Western blot

U251 glioma cells were cultured in a 6-well plate (6×10^5^ cells/well) overnight and transfected with PI3K p85 siRNA or control non-targeted siRNA (Guangzhou RIBOBIO, CO., LTD) with lipofectamine TM 2000 (Invitrogen) according to the manufacturer's instruction. Following transfection, cells were collected for Western blotting for p85 PI3K or treated with tamoxifen (5 µmol/L) for 12 h for flow cytometric analysis.

After different treatments, cells were washed three times with cold PBS and lysed in RIPA buffer on ice. Equivalent amounts of protein (20 µg) from the soluble fractions of the cell lysates were separated by 10% SDS–PAGE gel electrophoresis, and transferred to PVDF membrane (Amersham Biosciences, Little Chalfont, UK). After blocking with 5% nonfat milk in TBS containing 0.05% Tween 20 for 1 h, the membranes were incubated with appropriately diluted primary antibodies at 4°C overnight, and then probed with horseradish peroxidase–conjugated secondary antibodies at 1∶10,000 for 1 h at room temperature. Immunoreactive bands were detected by enhanced chemiluminescence and visualized with the Las4000 (GE Healthcare). The intensities of the blots were quantified by densitometry using MULTI GAUGE software according to manufacturer's instruction.

### Apoptosis assay

Hoechst 33342 staining: Nuclear DNA in treated cells contained in 24-well plates was visualized by staining with the DNA-specific dye Hoechst 33342 at a final concentration of 5 µg/ml. Cells were observed immediately with filters for blue fluorescence.

TUNEL staining: Treated cells grown on 8-well chamber slides were washed twice with PBS, and then fixed with 4% paraformaldehyde in PBS for 60 min at room temperature. After permeabilization with 0.1% Triton-X-100 (Sigma-Aldrich) for 2 min on ice, cells were stained with TUNEL reagent (Beyotime) for 60 min at room temperature in the dark for *in situ* apoptosis detection. Coverslips were mounted in Prolong Gold anti-fade reagent with DAPI (Molecular Probes) and inspected with a Zeiss 710 confocal microscope.

Flow Cytometric Analysis : After treatment, detached cells and harvested cells were labeled by annexin V-FITC/PI (propidium iodide) apoptosis detection kit (KeyGEN Biotech) according to the manufacturer's instruction. Apoptotic and necrotic cells were quantified by flow cytometer (Becton Dickinson, USA) and analysis by the CellQuest software. At least 10,000 cells were analyzed for each sample.

### Immunofluorescence staining

C6 glioma cells were treated with 20 µmol/L of LY294002, or 5 µmol/L of tamoxifen, or with combined 20 µmol/L of LY294002 and 5 µmol/L of tamoxifen for 30 min. Cells were fixed with 4% paraformaldehyde in PBS (15 min, room temperature), blocked for 30 min with10% normal goat serum in PBS containing 0.1% saponin, and incubated with primary antibodies diluted in blocking buffer (β-catenin, 1∶1000) for 1 h at room temperature. After washing with PBS, cells were incubated with Alexa 488 Fluor-labeled secondary antibodies (diluted 1∶1,000 in blocking buffer) for 1 h and washed with PBS. Coverslips were mounted in Prolong Gold anti-fade reagent with DAPI (Molecular Probes) and inspected with a confocal microscope (Zeiss 710).

### Preparation of Nuclear Extracts

Proteins in the nuclei of treated cells were extracted by Nuclear Extract Kit (Active Motif; USA) according to the manufacturer's instructions. Cells were washed twice with ice-cold PBS containing phosphatase inhibitors, and collected by gently scraping with cell lifters. Cell pellets were lysed in Hypotonic Buffer and followed by centrifugation at 5000× g for 5 min at 4°C. Nuclei were re-suspended in lysis buffer and vortexed for 10 seconds at the highest setting, followed by incubation for 30 minutes in ice on a rocking platform set at 150 rpm. After centrifugation at 14,000× g for 10 minutes at 4°C, the supernatants were collected as nuclear extracts.

### Quantitative Real-Time PCR

Quantitative Real-Time polymerase chain reaction (PCR) was conducted using standard procedures [16]. Total RNA was extracted using Trizol Reagent (Invitrogen, Carlsbad, CA, USA) and reverse-transcribed into cDNA (Takala) according to the manufacturer's instructions. Quantitative real time PCR was performed using SYBR Green II Master Mix (Takara, Dalian, China) in iCycler iQ thermocycler (Bio-Rad). The profile of thermal cycling consisted of initial denaturation at 95°C for 30 s, and 40 cycles at 95°C for 5 s and 60°C for 20 s. All primers used for Real-Time PCR analysis were synthesized by Invitrogen. The specificity of each primer pair was confirmed by melting curve analysis and agarose-gel electrophoresis. β-actin was used as internal control. Sequences of primers (5′ to 3′): for Survivin, forward- TTGCTCCTGCACCCCAGAGC, reverse-AGGCTCAGCGTAAGGCAGCC; for BCL-xL, forward-CTTCTCCTTTGGCGGGGCACTG, reverse-TCCACAAAAGTGTCCCAGCCGCC; for BCL-2, forward-CTCTCGTCGCTACCGTCGCG, reverse-AGGCATCCCAGCCTCCGTTATCC; for Mcl-1, forward-TGGAGGTGAACCCGACTTCCATG, reverse-TGGGGCTGGCTTGAGGTTCTCAA; for β-actin, forward-TCGTGCGTGACATTAAAGAG, reverse-ATTGCCGATGATAGTGATGACCT.

## Results

### Combination treatment with LY294002 and tamoxifen significantly inhibited the proliferation of glioma cells

**Figure 1:**
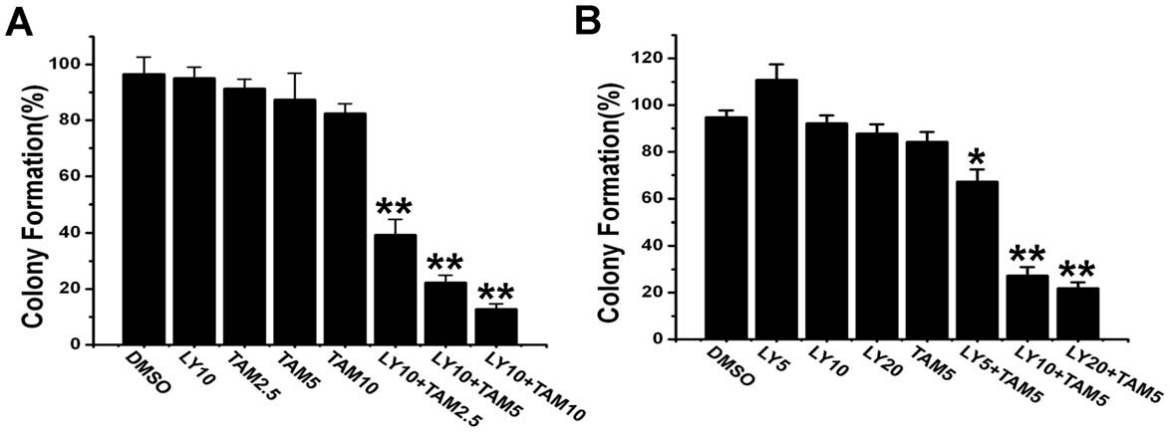
Colony formation assay with U251 cells receiving LY294002, tamoxifen, or the LY294002/tamoxifen combination. After 12 h treatment with LY294002 (LY), or/and tamoxifen (TAM), U251 cells were seeded into six-well plates (100 cells per well) and cultured for 14 days. The plates were stained with Giemsa, and the colonies were quantified. A. Effect of combination treatment with different concentration (2.5, 5, 10 µmol/L) of TAM and 10 µmol/L LY; B. Effect of combination treatment with different concentration of LY (5, 10, 20 µmol/L) and 5 µmol/L TAM.

The ability of combination treatment with LY294002 and tamoxifen to inhibit proliferation of U251 glioma cells was examined by plate colony formation assay. The number of colonies in controls and treated groups was counted and is summarized in Fig. 1. From these results it was evident that U251 cells receiving combination treatment exhibited much lower colony forming ability than those treated with LY294002 or tamoxifen alone. Tamoxifen (2.5, 5, 10 µmol/L) combined with 10 µmol/L LY294001 does-dependently inhibited colony formation (39%, 22%, 13% respectively, Fig. 1A). However, combination of LY294001 at 10 or 20 µmol/L with Tamoxifen (5 µmol/L) had a similar effect on colony formation (27%, 22% respectively, Fig. 1B).

### LY294002 enhanced the sensitization of glioma cells to tamoxifen-induced apoptosis

**Figure 2:**
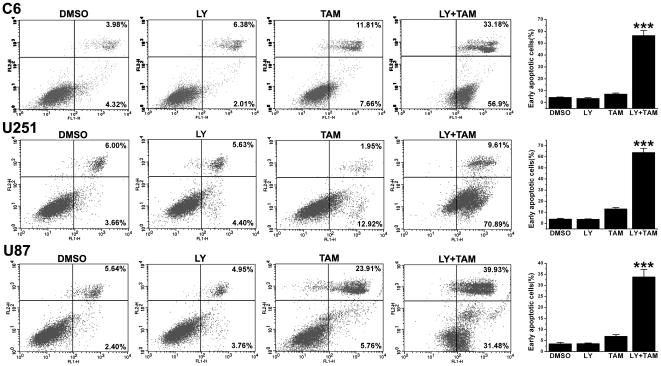
Flow cytometric analysis of apoptosis in C6, U251 and U87 glioma cells treated with LY294002 (LY), or/and tamoxifen (TAM). C6, U251 and U87 cells were treated with 10 µmol/L LY, or/and 5 µmol/L tamoxifen for 12 h, followed by Annexin V-FITC/propidium iodide staining. Columns representing the flow cytometry data presented in left. Data are means ±S.E.M. of values from three independent experiments; FL2-H, propidium iodide; FL1-H, Annexin V.

The effect of LY294002 on tamoxifen-induced apoptosis was evaluated by flow cytometric analysis, Hoechst staining, and TUNEL analysis. Annexin V-FITC/propidium iodide staining followed by flow cytometric analysis of apoptosis in C6, U251 and U87 glioma cells treated with LY294002 (10 µmol/L), or/and tamoxifen (5 µmol/L) revealed that the combination treatment significantly increased the early apoptotic cells (Annexin V+/PI−, Fig. 2).

**Figure 3:**
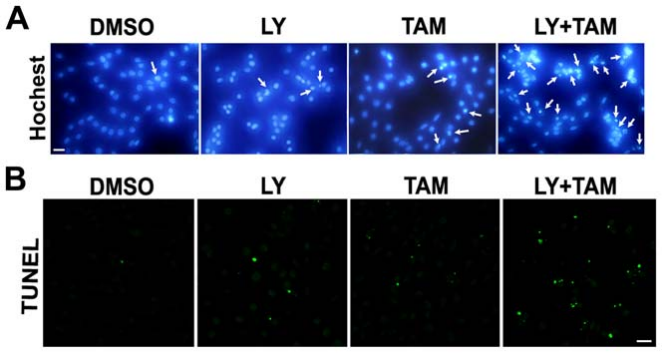
LY294002 enhanced the sensitization of C6 glioma cells to tamoxifen-induced apoptosis. C6 glioma cells were treated with PI3K inhibitor LY294002 (LY, 20 µmol/L), or/and tamoxifen (TAM, 5 µmol/L) for 24 h. The DMSO-treated cells were used as a control. After treatment, cells were subjected to the following experiments. A. Hochest 33342 staining; B. TUNEL staining. The apoptotic cells were marked by green fluorescence; Scale bar, 20 µm.

Apoptotic cells demonstrating nuclear condensation and DNA fragmentation can be detected by Hoechst 33342 staining and fluorescence microscopy. As illustrated in Fig. 3A, C6 glioma cells with combined treatment of LY294002 (20 µmol/L) and tamoxifen (5 µmol/L) for 24 h exhibited much more cells with condensed and fragmented nuclei than those treated with LY294002 or tamoxifen alone. Similar results were shown by TUNEL analysis (Fig. 3B). Fragmented DNA in nuclei was revealed as a green fluorescence signal. As shown in Fig. 1B, combination treatment with 20 µmol/L of LY294002 and 5 µmol/L of tamoxifen for 24 h showed predominantly increased DNA fragmentation compared with a single treatment with either LY294002 or tamoxifen.

### PI3K signaling pathway activation protected U251 cells from apoptosis induced by combination treatment of LY294002 and tamoxifen

**Figure 4:**
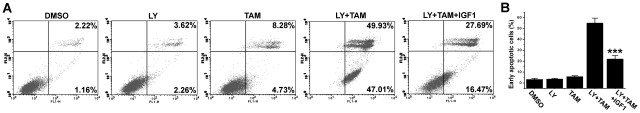
PI3K signaling pathway activation protected cells from apoptosis induced by combination treatment of LY294002 and tamoxifen. U251 cells were treated with 10 µmol/L LY294002 (LY), or/and 5 µmol/L tamoxifen (TAM), or combination of LY and TAM with additional IGF-1 (50 ng/ml) for 12 h, followed by Annexin V-FITC/propidium iodide staining. Columns representing the flow cytometry data presented in left. Data are means ±S.E.M. of values from three independent experiments; FL2-H, propidium iodide; FL1-H, Annexin V.

Since the inhibition of PI3K enhanced the sensitization of glioma cells to tamoxifen-induced apoptosis, we then studied whether the activation of PI3K signaling pathway could protect U251 cells from apoptosis induced by the combination treatment? As shown in Fig. 4, the early apoptotic cells were significantly increased by combination treatment with LY294002 (10 µmol/L) and tamoxifen (5 µmol/L) compared with single treatment; and Activation PI3K signaling pathway by IGF-1 (50 ng/ml) obviously decreased the early apoptotic cells induced by combination treatment (Fig. 2A and B).

### PI3K-P85 siRNA enhanced the sensitization of U251 glioma cells to tamoxifen -induced apoptosis

**Figure 5:**
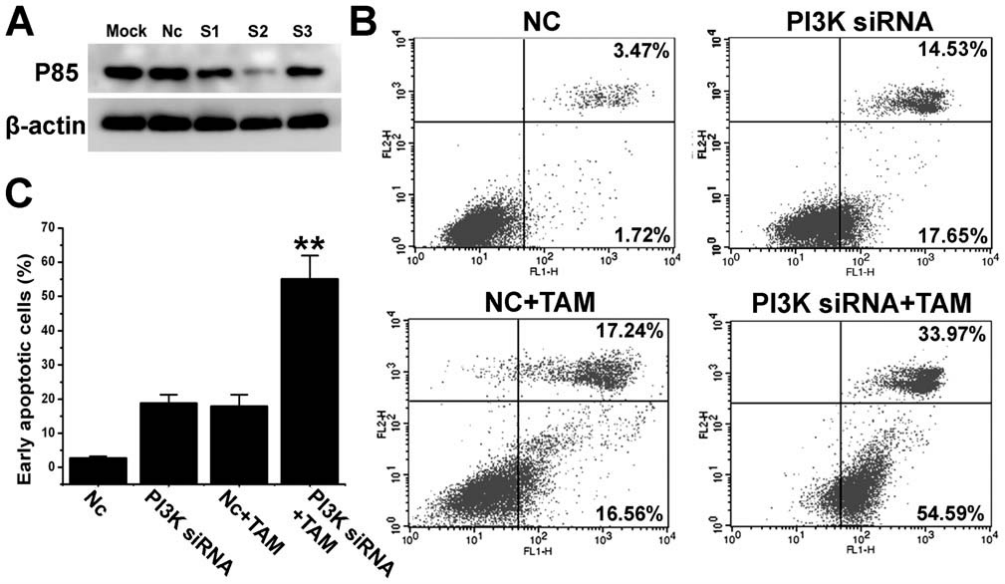
PI3K-P85 siRNA enhanced the sensitization of U251 glioma cells to tamoxifen -induced apoptosis. A. After 48 h transfection with non-targeted (Nc), or PI3K-P85 (S1,S2 or S3) siRNA, or without siRNA (Mock), total cell lysates (20 µg) were subjected to Western blot analysis. B. After 48 h transfection with non-targeted (Nc), or PI3K-P85 S2 siRNA, cells were treated without or with 5 µmol/L tamoxifen (TAM) for 12 h, followed by Annexin V-FITC/propidium iodide staining. C. Columns representing the flow cytometry data presented in B. Data are means ±S.E.M. of values from three independent experiments. FL2-H, propidium iodide; FL1-H, Annexin V.

To further confirm the involvement of PI3K signaling pathway in sensitization of glioma cells to tamoxifen-induced apoptosis, we then examined the effect of PI3K signaling pathway interference by short interfering RNA (siRNA) duplexes targeting the PI3K subunit p85. Three different siRNA duplexes were tested, and S2 had the highest efficiency (Fig. 5A) and was used for further experiments. Depletion p85 by S2 siRNA in U251 cells increased the early apoptotic cells (19%, Fig. 5B and C). This effect was much enhanced by 5 µmol/L tamoxifen treatment (55%, Fig. 5B and C). The result confirmed that the inhibition of PI3K signaling pathway could sensitize glioma cells to tamoxifen-induced apoptosis.

### Combination treatment with LY294002 and tamoxifen significantly decreased Akt^Ser473^ and GSK-3β^Ser9^ phosphorylation in C6 glioma cells

**Figure 6:**
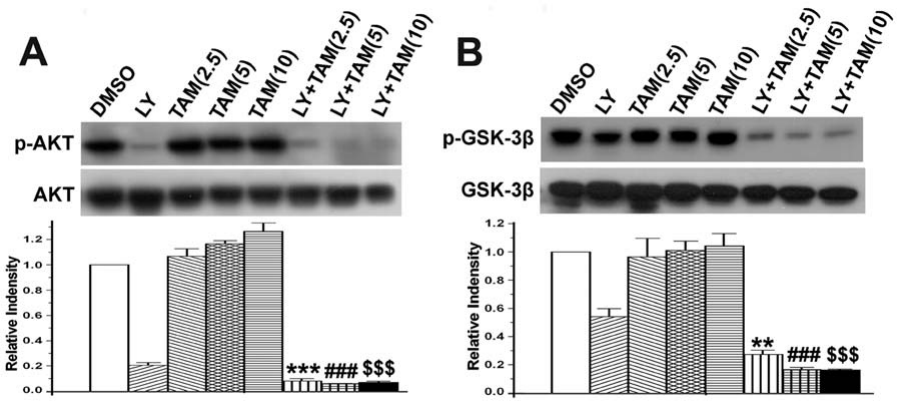
Combination treatment with LY294002 and tamoxifen remarkably decreased AKT and GSK-3β phosphorylation in C6 glioma cells. C6 glioma cells were treated with PI3K inhibitor LY294002 (LY, 20 µmol/L), or/and tamoxifen (TAM, 5 µmol/L) for 12 h. The DMSO-treated cells were used as a control. Proteins (20 ug) from total cell lysate were separated by SDS-PAGE gel electrophoresis, and immunoblotted with antibodies against Akt(pan), phosphorylated Akt^Ser473^ (A), GSK-3β(pan), or phosphorylated GSK-3β^Ser9^ (B), and followed by densitometry quantification. Data are means±S.E.M. of values from three independent experiments. **p<0.01, ***p<0.001 *vs* single treatment with 2.5 µmol/L of tamoxifen; ### *p*<0.001 *vs* single treatment with 5 µmol/L of tamoxifen; $$$ *p*<0.001 *vs* single treatment with 10 µmol/L of tamoxifen.

To understand the molecular mechanisms by which LY294002 combined with tamoxifen resulted in increased apoptosis of C6 glioma cells, the levels of phosphorylated Akt^Ser473^ and GSK-3β^Ser9^ were compared after 12 hours single or combined treatment with LY (20 µmol/L) or/and tamoxifen (2.5, 5, 10 µmol/L). As shown in Fig. 6, both phosphorylated Akt^Ser473^ and GSK-3β^Ser9^ were decreased by LY294002 treatment, but were not affected by single tamoxifen treatment. After a combination treatment with LY294002 and tamoxifen, both phosphorylated Akt^Ser473^ and GSK-3β^Ser9^ were significantly decreased (Fig. 6). Compared with LY294002 single treatment, combination treatment significantly reduced GSK-3β^Ser9^ phosphorylation in a dose (tamoxifen)-dependent manner (Fig. 6B, p<0.01).

### Time-course changes of phosphorylated Akt^Ser473^ and GSK-3β^Ser9^ after single or combined treatment with LY294002 or/and tamoxifen

**Figure 7:**
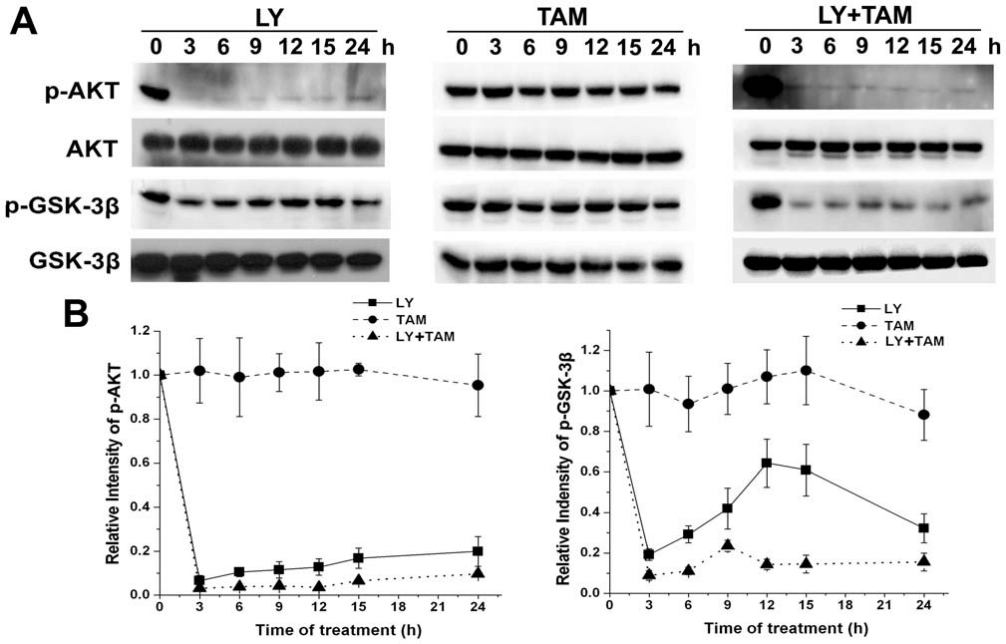
Time-course changes of phosphorylated Akt^Ser473^ and GSK-3β^Ser9^ after 24 h of single or combined treatment with LY294002 or/and tamoxifen. C6 glioma cells were treated with LY294002 (LY, 20 µmol/L), or/and tamoxifen (TAM, 5 µmol/L) for indicated times. The DMSO-treated cells were used as a control. Proteins (20 ug) from total cell lysate were separated in SDS-PAGE gel, and immunoblotted with antibodies against Akt(pan), phosphorylated Akt^Ser473^, GSK-3β(pan), or phosphorylated GSK-3β^Ser9^ (A), followed by densitometry quantification (B). Data are means ±S.E.M. of values from three independent experiments.

To evaluate the effects of combined treatment on phosphorylated Akt^Ser473^ and GSK-3β^Ser9^, immunoblotting was performed with specific antibodies against phosphorylated Akt^Ser473^ and GSK-3β^Ser9^ using extracts from C6 glioma cells treated with LY294002 (20 µmol/L), or tamoxifen (5 µmol/L), or with a combined treatment of LY294002 (20 µmol/L) and tamoxifen (5 µmol/L) for various times (3, 6, 9, 12, 15 and 24 h). Our results showed that although LY294002 single treatment and combined treatment could keep phosphorylated Akt^Ser473^ at lower levels within 24 h (Fig. 7), combined treatment was much more efficient in inhibiting GSK-3β^Ser9^ phosphorylation (Fig. 7). Compared with LY294002 single treatment, cells with combined treatment revealed a significant reduction of phosphorylated GSK-3β^Ser9^ within 24 h (Fig. 7B). Tamoxifen single treatment had no obvious effects on phosphorylated Akt^Ser473^ and GSK-3β^Ser9^ in the observed period (Fig. 7).

**Figure 8:**
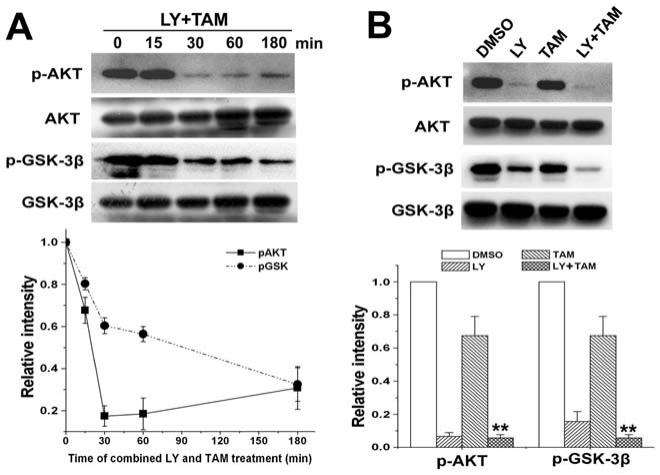
Combination treatment of LY294002 and tamoxifen rapidly decreased Akt and GSK-3β phosphorylation in C6 glioma cells. A. C6 glioma cells were treated with combined LY294002 (20 µmol/L) and tamoxifen (5 µmol/L) for indicated times. The DMSO-treated cells were used as a control. Proteins (20 ug) from total cell lysate were separated in SDS-PAGE gel, and immunoblotted with antibodies against total Akt(pan), phosphorylated Akt^Ser473^, GSK-3β(pan), or phosphorylated GSK-3β^Ser9^, and followed by densitometry quantification. Data are means ±S.E.M. of values from three independent experiments. B. C6 glioma cells were treated with LY294002 (LY, 20 µmol/L), or/and tamoxifen (TAM, 5 µmol/L) for 30 min. The DMSO-treated cells were used as a control. Proteins (20 ug) from total cell lysate were subjected to SDS-PAGE gel electrophoresis, and immunoblotted with antibodies against Akt(pan), phosphorylated Akt^Ser473^, GSK-3β(pan), or phosphorylated GSK-3β^Ser9^, and followed by densitometry quantification. Data are means ±S.E.M. of values from three independent experiments. **p<0.01 *vs* single treatment with 5 µmol/L of tamoxifen.

Because phosphorylation of the proteins occurred immediately upon drug treatment, we then studied the changes of phosphorylated Akt^Ser473^ and GSK-3β^Ser9^ after combined treatment at shorter times (15, 30, 60, 180 min). As shown in Fig. 4A, phosphorylated Akt^Ser473^ was rapidly decreased after 30 min of combined treatment, and then slightly increased following the time of combined treatment (Fig. 8A). Interestingly, phosphorylated GSK-3β^Ser9^ was reduced by combined treatment in a time-dependent manner (Fig. 8A). Comparative studies showed that a combination treatment for 30 min reduced the phosphorylated GSK-3β^Ser9^ level more effectively than LY294002 or tamoxifen single treatment (Fig. 8B).

### Effect of LY294002 pretreatment combined with tamoxifen on the nuclear translocation of β-catenin in C6 glioma cells

**Figure 9:**
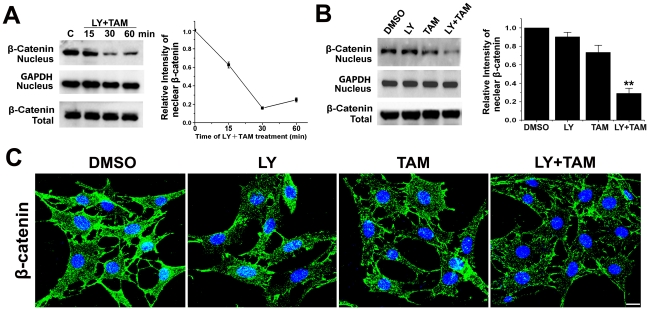
Effect of combined treatment with LY294002 and tamoxifen on the nuclear translocation of β-catenin in C6 glioma cells. A. C6 glioma cells were treated with combined LY294002 (20 µmol/L) and tamoxifen (5 µmol/L) for indicated times. The DMSO-treated cells were used as a control. The levels of β-catenin or GAPDH (loading control) in nuclear extracts were analysed by Western blotting and quantified by densitometry. Data are means ±S.E.M. of values from three independent experiments. B. C6 glioma cells were treated with 20 µmol/L of LY294002 (LY), or/and 5 µmol/L of tamoxifen (TAM) for 30 min. The DMSO-treated cells were used as a control. The levels of β-catenin or GAPDH (loading control) in nuclear extracts were analysed by Western blotting and quantified by densitometry. Data are means ±S.E.M. of values from three independent experiments. **p<0.01 *vs* single treatment with 5 µmol/L of tamoxifen. C. C6 glioma cells were treated with 20 µmol/L of LY294002 (LY), or/and 5 µmol/L of tamoxifen (TAM) for 30 min. The cells were fixed and immunereacted with anti-β-catenin antibody followed by incubation with Alexa 488-labeled secondary antibody. Images were taken by a confocal microscope (Zeiss 710). Scale bar, 20 µm.

To detect whether increased apoptosis of C6 glioma cells caused by combined treatment of LY294002 and tamoxifen was modulated by GSK-3β/β-catenin signaling pathway, nuclear β-catenin was measured by western-blotting and confocal microscopy. As shown in Fig. 9A, nuclear β-catenin level was rapidly reduced by a combination treatment for 30 min, then slightly recovered after 60 min. Compared with LY294002 or tamoxifen single treatment, a combined treatment could significantly inhibit the translocation of β-catenin into the nucleus (Fig. 9B). Confocal microscopy also confirmed much less nuclear staining of β-catenin in cells with combined treatment than those with LY294002 or tamoxifen single treatment (Fig. 9C).

### Effect of combination treatment with LY294002 and tamoxifen on the expression of anti-apoptotic genes

**Figure 10:**
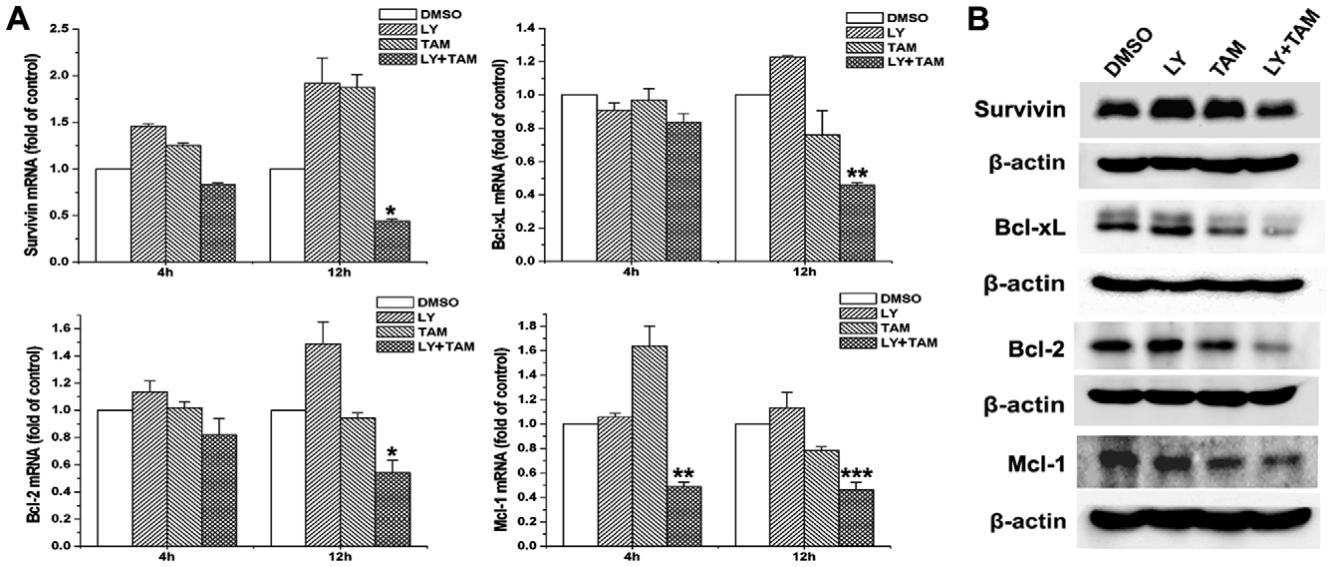
Effect of combination treatment with LY294002 and tamoxifen on the expression of anti-apoptotic genes. **A.** C6 glioma cells were treated with 20 µmol/L of LY294002 (LY), or/and 5 µmol/L of tamoxifen (TAM) for 4 or 12 h. The DMSO-treated cells were used as a control. Total RNA was extracted and the mRNA levels of Survivin, Bcl-XL, Bcl-2 and Mcl-1 were analysised by quantitative real-time PCR. Data were presented as fold change over control group. Data are means ±S.E.M. of values from three independent experiments. *p<0.05, ** *p*<0.01, ***p<0.001 *vs* control. B. C6 glioma cells were treated with 10 µmol/L of LY294002 (LY), or/and 5 µmol/L of tamoxifen (TAM) for 24 h. The DMSO-treated cells were used as a control. Total cell lysates were extracted and the levels of Survivin, Bcl-XL, Bcl-2 and Mcl-1 were analysed by Western blotting.

Because combined treatment could inhibit the translocation of β-catenin into nucleus, we therefore investigated the mRNA levels of anti-apoptotic genes that were possibly regulated by GSK-3β/β-catenin signaling pathway, such as survivin, Bcl-XL, Bcl-2, and Mcl-1. Our results showed that LY294002 single treatment for 12 h resulted in a remarkable increase of Bcl-2 and survivin mRNA, which was up-regulated after only 4 h of LY294002 treatment; A slight increase of Bcl-XL and Mcl-1 mRNA was also observed after 12 h of LY294002 single treatment, but failed to reach statistic significance (Fig. 10A). Tamoxifen single treatment had no effect on Bcl-2 mRNA, but strongly induced mRNA expression. The change of Mcl-1 mRNA by tamoxifen single treatment was bidirectional: increased after 4 h of treatment and decreased after 12 h of treatment. A Slight decrease of Bcl-XL mRNA was also seen after 12 h of tamoxifen treatment (Fig. 10A). The message RNA levels in all four genes were significantly reduced by 12 h of combination treatment. Interestingly, the effect on Mcl-1 mRNA was especially strong. An obvious decrease of Mcl-1 mRNA was found after only 4 h of combined treatment (Fig. 10A). Western blot analysis revealed that the changes of survivin, Bcl-XL, Bcl-2, and Mcl-1 protein in C6 glioma cells treated with 10 µmol/L of LY294002, or/and 5 µmol/L of tamoxifen for 24 h were similar as the changes of mRNA (Fig. 10B).

## Discussion

Collective data on the genetic and cellular mechanisms of glioma survival, proliferation and invasion indicated that the malignant tumor phenotype results from the dysfunction of multiple interrelated growth-regulatory pathways. One of the most important effectors of growth factor receptor signaling is the PI3K-Akt pathway, which is activated by multiple growth factor receptors and functions as a central signaling node for the activation of downstream effectors. In malignant gliomas, overexpression and constitutive activation of growth factor receptors, such as EGF receptor and IGF-1 receptor, and inactivation of pathway-controlling tumor suppressor genes such as PTEN, lead to overactivation of PI3K-AKT signaling and its downstream signal transduction pathways. The overactivated PI3K/Akt pathway not only plays a central role in maintaining the aggressive malignant phenotype of gliomas, but also mediates treatment resistance by promoting cellular survival and inhibiting the induction of apoptosis. It, therefore, became an attractive target for the treatment of malignant glioma [17].

However, single-targeted therapeutics against PI3K/AKT pathway has demonstrated only modest clinical benefits, which may be attributed to high resistance caused by multiple concomitantly activated pathways in gliomas [17]. Selecting appropriate combinations of anticancer agents that can exert synergistic cytotoxic interactions has been widely adopted and utilized in preclinical and clinical studies [18]. In the present study, we compared the proapoptotic effect of single-treatment with LY294002, a specific PI3K inhibitor, or tamoxifen, a traditional cytotoxic chemotherapic agent, with a combination treatment of those two agents. Our results revealed that LY294002 significantly enhanced the sensitization of tamoxifen-induced apoptosis in C6, U251 and U87 glioma cells. To understand the underlying molecular mechanisms, we then investigated the activation of downstream effectors of the PI3K signaling pathway. High levels of phosphorylated Akt^Ser473^ was found in C6 glioma cells. Moreover, GSK-3β, a protein that is constitutively active in resting cells, was functionally inactivated by phosphorylation. This observation is similar to the findings in human glioma [19]. Although LY294002 treatment could rapidly decrease the phosphorylation of Akt^Ser473^, and maintain it at low levels within 24 h, the phosphorylation of GSK-3β^Ser9^ remained at a higher levels (Fig. 7). Both phosphorylated Akt^Ser473^ and GSK-3β^Ser9^ remained at high levels within 24 h of tamoxifen single treatment (Fig. 8). Interestingly, combined treatment with LY294002 and tamoxifen showed an even lower level of phosphorylated Akt^Ser473^ than LY294002 single treatment. Phosphorylation of GSK-3β^Ser9^ was quickly down-regulated and continued to be inhibited within 24 h of combination treatment (Fig. 7). Those results indicated that the GSK-3β signaling pathway could be responsible for synergistic cytotoxic effect of LY294002 and tamoxifen combination treatment.

GSK-3β, a multifunctional serine-threonine protein kinase, regulates numerous signaling pathways involved in diverse cellular processes, including metabolism, cell cycle control, proliferation, differentiation and apoptosis. Recently, the pathologic role of GSK3β in malignant brain tumor was investigated, and it was found that it could protect glioblastoma cells from apoptosis by promoting survival and proliferation [20]. GSK3β also plays an important role in regulating differentiation and growth arrest in malignant glioma cells and glioblastoma [21], [22]. Specific pharmacologic GSK-3β inhibitors and siRNA knockdown of GSK-3β reduced glioma cell motility [23]. GSK-3β, therefore, was considered as a potential therapeutic target in malignant glioma. As a well-established component of the Wnt/β-catenin signalling pathway, GSK-3β directly determines the stabilization of β-catenin by phosphorylation. In the absence of Wnt activating signals, β-catenin is sequestered in the cytoplasm by a multiprotein complex, which includes GSK-3β. In this state, β-catenin is phosphorylated by GSK-3β, which targets it for ubiquitination and proteolytic degradation [24]. Activation of Wnt signaling inhibits the formation of the multiprotein complex, and the phosphorylation of β-catenin by GSK-3β. As a result, there is an accumulation of stabilized hypophosphorylated β-catenin, which then translocates to the nucleus and associates with transcription factors of the Lef/Tcf family to initiate the expression of a broad range of genes, such as c-myc, cyclin D1,and survivin [25]. Aberrant activation of Wnt/β-catenin signaling was found to be involved in glioma development and progression [26]. Knockdown of β-catenin by siRNA in human U251 glioma cells inhibited cell proliferation and invasive ability, and induced apoptotic cell death [27]. Recent evidence indicated that β-catenin transcriptional-promoting activity is an important way to induce a state of apoptosis resistance [28], [29]. In the present study, we showed a significant decrease of nuclear β-catenin by combination treatment with LY294002 and tamoxifen. However, only a slight decrease was found in the cells of single treatment with LY294002 or tamoxifen. These data indicate that the synergistic apoptosis-inducing effect of LY294002 and tamoxifen combination treatment could be attributed to the release of apoptotosis resistance in β-catenin signaling.

Antiapoptotic genes are the key factors in the development of apoptosis resistance and are believed to play a crucial role in chemotherapeutic drugs resistance. The inhibition of β-catenin signaling by LY294002 and tamoxifen combination treatment led us to study the changes of an important pro-survival gene, Survivin. Survivin is controlled via the β-catenin-Tcf/Lef pathway, and has attracted a great deal of interests because of its up-regulation in most human tumors for tumor survival [30], [31]. Clinical studies reported that invariable over expression of Survivin is associated with resistance to chemotherapy or radiation therapy, and is linked to poor prognosis [32]. Our results showed that Survivin mRNA was significantly decreased by LY294002 and tamoxifen combination treatment in response to the inhibition of β-catenin signaling. However, a single-treatment with LY294002 or tamoxifen resulted in a remarkable up-regulation of Survivin, which may partly explain their weak effect on apoptosis induction.

Other important antiapoptotic genes are Bcl-2 family members Bcl-2, Bcl-xL, and Mcl-1. These were considered key regulators of apoptosis [33]. Upregulated expression of antiapoptotic proteins of Bcl-2 family members commonly occurs in human malignancies, and is related with disease maintenance and progression, chemotherapy resistance, and poor clinical prognosis [34]. In the present study, the mRNA levels of all three antiapoptotic Bcl-2 genes Bcl-2, Bcl-xL, and Mcl-1 were significantly reduced by combination treatment of LY294002 and tamoxifen. The decrease in Mcl-1 mRNA was rapid and was the strongest. Mcl-1 was initially reported in differentiating myeloid cells [35]. As a pro-survival protein, high levels of Mcl-1 were detected in human malignancies such as in prostate, breast, colon and lung [36], and appear to be a factor in the resistance of some cancer types to conventional cancer therapies [37]. Unlike the other members of the Bcl-2 family, Mcl-1 has a very short half-life, which allows for either the rapid induction or elimination in response to cell survival or cell death events [38]. Therefore, through inhibition of its anti-apoptotic function by down-regulation, its expression could prominently increase chemotherapeutic sensitization and make Mcl-1-dependent cells more susceptible to apoptosis [34], [37], [39]. It has been reported that the activity of GSK-3β was required for Mcl-1 degradation, which is an essential mechanism for GSK-3β-induced apoptosis and contributes to GSK-3β-mediated tumor suppression and chemosensitization [40], [41]. In accordance with those findings, our results indicated sensitization of glioma cells to combination treatment with Ly294002 and tamoxifen benefited from its inhibitory effects on antiapoptotic gene expression.

Taken together, our data demonstrate that a specific PI3K inhibitor LY294002 significantly enhanced the sensitization of glioma cells to tamoxifen-induced apoptosis. We also showed that the GSK-3β/β-catenin signaling pathway and related antiapoptotic genes play critical roles in regulating cell survival or cell death events in malignant glioma cells, and are the key factors in the development of apoptosis resistance. The inhibition of β-catenin signaling pathway and down-regulated expression of antiapoptotic genes is due to increased GSK-3β activity that might be responsible for synergistic cytotoxic effect of LY294002 and tamoxifen.
